# Bilirubin Levels as Potential Indicators of Disease Severity in Coronavirus Disease Patients: A Retrospective Cohort Study

**DOI:** 10.3389/fmed.2020.598870

**Published:** 2020-11-09

**Authors:** Zeming Liu, Jinpeng Li, Wei Long, Wen Zeng, Rongfen Gao, Guang Zeng, Danyang Chen, Shipei Wang, Qianqian Li, Di Hu, Liang Guo, Zhengwei Li, Xiaohui Wu

**Affiliations:** ^1^Department of Plastic Surgery, Zhongnan Hospital of Wuhan University, Wuhan, China; ^2^Department of Thyroid and Breast Surgery, Zhongnan Hospital of Wuhan University, Wuhan, China; ^3^Department of Ophthalmology, Zhongnan Hospital of Wuhan University, Wuhan, China; ^4^Department of Rheumatology and Immunology, Tongji Medical College, Tongji Hospital, Huazhong University of Science and Technology, Wuhan, China; ^5^Department of Urology, Zhongnan Hospital of Wuhan University, Wuhan, China; ^6^Department of Neurosurgery, Zhongnan Hospital of Wuhan University, Wuhan, China

**Keywords:** COVID-19, bilirubin, length of hospitalization, mortality, disease severity

## Abstract

**Objectives:** The coronavirus disease (COVID-19) pandemic has caused a large number of deaths. Some patients with severe or critical COVID-19 have been observed to have elevated bilirubin levels. Studies on the association of bilirubin level and mortality in patients with COVID-19 are limited. This study aimed to examine the role of bilirubin levels in COVID-19 severity and mortality.

**Methods:** A retrospective cohort study was conducted in patients hospitalized with COVID-19 in Leishenshan Hospital in Wuhan, China. Cox regression analyses and logistic regression analyses were conducted to investigate the risks for mortality and disease severity, respectively. Kaplan–Meier analyses with log-rank tests were performed to assess the association between bilirubin level and survival.

**Results:** In total, 1,788 patients with COVID-19 were included in the analysis. 5.8% (4/69) of patients in the elevated serum total bilirubin (STB) group died, compared to 0.6% (11/1,719) of patients in the non-elevated STB group. The median alanine aminotransferase (ALT) and aspartate aminotransferase (AST) activities in the elevated STB group were 29 U/L [interquartile range (IQR): 16–45 U/L] and 22 U/L (IQR: 13–37 U /L), respectively, which were significantly higher than the median ALT (median: 23, IQR: 15–37) and AST (median: 20, IQR: 16–26) activities in the non-elevated STB group (both *p* < 0.05). Patients with an elevated STB level showed increased mortality [hazard ratio (HR): 9.45, *P* = 0.002], elevated conjugated bilirubin (CB) levels (HR: 4.38, *P* = 0.03), and an elevated ratio of CB to unconjugated bilirubin (UCB, CB/UCB) (HR: 2.49, *P* = 0.01). CB/UCB was positively correlated with disease severity (odds ratio: 2.21, *P* = 0.01).

**Conclusions:** COVID-19 patients with elevated STB and CB levels had a higher mortality, and CB/UCB was predictive of disease severity and mortality. Thus, it is necessary to pay special attention to COVID-19 patients with elevated bilirubin levels in clinical management.

## Introduction

In December 2019, coronavirus disease (COVID-19), caused by severe acute respiratory syndrome (SARS) coronavirus 2 (SARS-CoV-2), emerged in Wuhan, China (1, 2). Soon thereafter, it became a pandemic. More than 200 countries and territories have reported confirmed cases. However, there are currently no specific drugs for treatment. As of October 10, 2020, over 36 million people had been infected with SARS-CoV-2 and over one million people had died of COVID-19 worldwide (3).

Lungs are recognized as the primary target organs for COVID-19 (4). However, COVID- 19 patients frequently show evidence of damage to other organs. Furthermore, those with pre-existing liver disease or newly occurred evidence of liver injury have an increased likelihood of a poor prognosis (5, 6). A number of studies have suggested that liver disease is one of the most common comorbidities of COVID-19 patients and a number of patients infected with SARS-CoV-2 can develop different degrees of liver injury (7, 8). To monitor liver damage, bilirubin levels are a universally accepted marker.

Most studies on COVID-19 are descriptive and focus on its epidemiological and clinical characteristics (9–11). Studies on the risk factors associated with mortality in patients with COVID-19 are controversial. Elevation of bilirubin levels has been observed in some COVID-19 patients with severe or critical disease. Previous studies have proposed a link between bilirubin levels and disease severity, but they have had relatively small sample sizes and have not explored the relationship between bilirubin levels and the survival of patients with COVID-19 (8, 12, 13). Therefore, we aimed to determine the association between bilirubin levels and disease severity and mortality in patients with COVID-19 based on a large sample.

## Patients and Methods

### Setting

The Leishenshan Hospital, Wuhan, China, was rapidly built as a designated hospital for patients with COVID-19. The hospital contains 1600 beds. On February 8, 2020, the Leishenshan Hospital treated its first patients. By April 15, 2020, it had served its purpose and was officially closed. During the time that it was in operation, a total of 1,880 patients with confirmed COVID-19 were admitted.

### Study Design and Participants

This retrospective study was approved by the Research Ethics Commission of Zhongnan Hospital of Wuhan University (No. 2020074), and considering the rapid spread of COVID-19, the requirement for patient consent was waived by the ethics commission. Patients without data on serum total bilirubin (STB) levels were excluded. We included 1,788 patients: 1,785 patients admitted from February 8 to March 18 and 3 patients with a missing admission date. The follow-up period lasted until April 14, 2020.

### Data Collection

We obtained patient information including that of demographic and clinical characteristics, laboratory findings, computed tomography (CT) images, clinical management, and outcomes from the electronic medical record system. All information was typed in a pre-designed data collection form. All data were independently examined by three investigators to ensure data accuracy.

### Definitions

Patients enrolled were divided into elevated STB group and non-elevated STB group according to their STB levels on admission. The reference range for the physiological STB concentration was 5–21 μmol/L. The cut-point was the upper limit of reference range. We regarded the survival (alive or dead) and disease severity of patients with COVID-19 as the primary outcome variables. Disease severity was classified as Mild, Common, Severe, or Critical based on the seventh version of the guidelines for the diagnosis and treatment of COVID-19 published by the National Health Commission of China (14). Mild disease was characterized by mild clinical symptoms with no findings of pneumonia on imaging. Common disease was characterized by fever, respiratory symptoms, and other symptoms, with signs of pneumonia revealed on imaging. Severe disease was characterized by the presence of any one of the following factors: (1) shortness of breath or a respiratory rate of ≥30 beat per minute (BPM); (2) oxygen saturation of ≤ 93% at rest; (3) arterial partial pressure of oxygen (PaO_2_)/fraction of inspired oxygen (FiO_2_) ≤ 300 mmHg; and (4) a lesion that has progressed by more than 50% within 24–48 h as seen on pulmonary imaging. Critical disease was characterized by the presence of any one of the following factors: (1) respiratory failure requiring mechanical ventilation; (2) shock state; and (3) organ failure requiring intensive care. In one patient, the highest level of severity at hospitalization was mild disease; thus, we classified the patient as having both mild and common disease. CT findings were also a vital outcome in our study. Two experienced radiologists assessed all chest CT images and reached a consistent rating after discussion. In the early stage, chest CT images mainly showed ground-glass opacities (GGO) and reticulation or cord changes. Consolidation was a manifestation observed in the progression stage, and pleural effusion was rarely observed (15). Score 1 was calculated according to whether the CT images showed GGO, reticulation or cord changes, consolidation, and pleural effusion. One point was assigned for each presentation, and the sum of these points was considered Score 1. The area of lung lobe involvement was reflected by Score 2: no involvement, 0; <25%, 1; 26–50%, 2; 51–75%, 3; and 76–100%, 4. The total score was the sum of scores 1 and 2. The days of CT scores was calculated as the duration from onset to CT scans.

### Statistical Analysis

We used median (interquartile range) and frequency (percentage) to present continuous and categorical variables, respectively. To discern the differences in baseline characteristics between the groups with non-elevated STB levels and elevated STB levels, continues variables were analyzed by the Mann-Whitney test and Chi-square test, and Fisher's exact test was adopted for categorical variables accordingly. Univariate and multivariate Cox regression analyses were conducted to investigate the risk of survival and disease severity in patients with COVID-19 according to STB levels. Univariate and multivariate logistic regression analyses were performed to identify the relationship between disease severity and the ratio of conjugated bilirubin to unconjugated bilirubin (CB/UCB). The survival of patients with COVID-19 according to STB status was assessed using Kaplan–Meier analyses and log-rank tests. A receiver operating characteristic (ROC) curve was used to determine the relationship between CB/UCB and disease severity or length of hospitalization. Curve fitting analyses were performed to detect the relationship between days from onset and CT scores. All statistical analyses were performed using SPSS, version 22.0 (IBM Corp., Armonk, NY, United States). Statistical significance was defined using a two-sided *P* < 0.05.

## Results

### Participant Baseline Characteristics

This study cohort included 1,788 patients with COVID-19 who were admitted to the Leishenshan Hospital. Among the 1,788 patients, 69 were assigned to the elevated STB group [median age: 56 years, interquartile range (IQR): 46–67 years; 68.1% male] and the remaining 1,719 were assigned to the non-elevated STB group [median age: 59 (IQR: 49–68) years; 47.7% male]. The demographic and clinical features are shown in Table 1. The elevated STB group included a higher proportion of patients who required tracheal intubation and extracorporeal membrane oxygenation (ECMO) than the STB normal or decreased group (*P* < 0.001). Moreover, the elevated STB group also had a higher rate of critical disease (*P* = 0.04). Laboratory testing results are shown in Table 2. Levels of interleukin-6, procalcitonin, alanine aminotransferase (ALT), aspartate aminotransferase (AST), creatinine, and D-dimer and international normalized ratio (INR), prothrombin time, and platelet count all differed significantly according to STB level. The median ALT and AST activities in the elevated STB group were 29 U/L (IQR: 16–45) and 22 (IQR: 13–37), respectively, which were significantly higher than the median activities of ALT (median: 23, IQR: 15–37) and AST (median: 20, IQR: 16–26) in the non-elevated STB group (both *p* < 0.05). The underlying comorbidities are presented in Table 3. The number of patients with cardiovascular diseases, malignancies, and digestive system diseases was higher in the elevated STB group than in the non-elevated STB group. However, these differences were not statistically significant. Patients with elevated CB levels had significantly high rates of having concurrent cardiovascular diseases (*p* = 0.01) and digestive system diseases (*p* = 0.03).

**Table 1:** Demographic and clinical features of 1,788 patients with COVID-19.

**Covariate**		**Total (*n* = 1,788)**	**Non-elevated STB group(*n* = 1,719)**	**Elevated STB group (*n* = 69)**	***P*-value**
Age, year		59 (49–68)	59 (49–68)	56 (46–67)	0.38
Sex					0.001
Female	936 (52.3)	914 (52.3)	22 (31.9)	
Male	852 (47.7)	805 (47.7)	47 (68.1)	
Disease severity on admission					0.07
Mild	670 (37.5)	653 (38)	17 (24.6)	
Common	808 (45.2)	768 (44.7)	40 (58)	
Sever	285 (15.9)	275 (16)	10 (14.5)	
Critical	25 (14)	23 (1.3)	2 (2.9)	
The highest level of severity at hospitalization					0.04
Mild and Common	931 (52.2)	900 (52.5)	31 (45.6)	
Sever	804 (45.1)	772 (45)	32 (47.1)	
Critical	48 (2.7)	43 (2.5)	5 (7.4)	
The highest level of oxygen support					<0.001
Low flow oxygen therapy	257 (82.9)	245 (83.3)	12 (75)	
High flow oxygen therapy	47 (15.2)	45 (15.3)	2 (12.5)	
Tracheal intubation	5 (1.6)	4 (1.4)	1 (6.3)	
ECMO	1 (0.3)	0	1 (6.3)	
Symptoms when admitted to the hospital					
Fever or Myalgia	621 (79)	597 (79.7)	24 (64.9)	0.03
Respiratory system symptoms	635 (80.8)	605 (80.8)	30 (81.1)	0.96
Digestive system symptoms	82 (10.4)	79 (10.5)	3 (8.1)	0.64
Nervous system symptoms	27 (3.4)	26 (3.5)	1 (2.7)	0.80
Other system symptoms	26 (3.3)	24 (3.2)	2 (5.4)	0.46
Antiviral therapy		869 (54.2)	840 (48.9)	29 (42.0)	0.16
Antibiotic therapy		521 (29.1)	500 (29.1)	21 (30.4)	0.12
The appliance of vitamin C		248 (13.9)	236 (13.7)	12 (17.4)	
Traditional Chinese medicine therapy		1,533 (85.7)	1,478 (82.5)	55 (79.7)	
Anticoagulation treatment		131 (7.3)	120 (7.0)	11 (15.9)	0.01
Use of corticosteroid		106 (5.9)	101 (5.9)	5 (7.2)	0.60
Use of antimalarial		139 (7.8)	135 (7.9)	4 (5.8)	0.05
Length of hospitalization, day		18 (13–24)	18 (13–24)	19 (14–24)	0.78
CT scores					1.00
0–4	78 (39.8)	75 (40.1)	3 (33.3)	
5–7	118 (60.2)	112 (59.9)	6 (66.7)	

**Table 2:** Laboratory results of 1,788 patients with COVID-19.

**Covariate**	**Total (*n* = 1,788)**	**Non-elevated STB group (*n* = 1,719)**	**Elevated STB group (*n* = 69)**	***P*-value**	**Reference range**
Interleukin-6, pg/mL	1.5 (1.5–4.0)	1.5 (1.5–3.75)	5.7 (1.5–20.9)	<0.001	0–7.0
Procalcitonin, ng/mL	0.04 (0.03–0.05)	0.04 (0.03–0.05)	0.05 (0.03–0.10)	<0.001	<0.05
Alanine aminotransferase, U/L	23 (15–37)	23 (15–37)	29 (16–45)	0.04	9–50
Aspartate aminotransferase, U/L	20 (16–27)	20 (16–26)	22 (17–37)	0.02	15–40
Albumin, g/L	37.7 (35.0–40.0)	37.7 (35.0–39.9)	38.5 (35.1–40.6)	0.46	40.0–55.0
Creatinine, μmol/L	64.3 (54.5–74.2)	64.1 (54.3–76.1)	68.2 (60.9–79.4)	0.01	64.0–104.0
Ureanitrogen, mmol/L	4.8 (3.9–5.8)	4.8 (3.9–5.8)	5.1 (4.1–6.4)	0.11	2.8–7.6
INR	1.0 (0.9–1.0)	1.0 (0.9–1.0)	1.0 (1.0–1.1)	<0.001	0.8–1.3
Prothrombin time, s	11.3 (10.9–11.8)	11.3 (10.9–11.7)	11.7 (11.1–12.4)	<0.001	9.4–12.5
Thrombin time, s	17.6 (17.0–18.4)	17.7 (17.0–18.4)	17.4 (16.9–18.6)	0.44	10.3–16.6
Activated partial thromboplastin time, s	27.2 (24.6–30.4)	27.2 (24.6–30.4)	27.3 (24.1–30.1)	0.82	25.1–36.5
Fibrinogen, g/L	3.0 (2.5–3.7)	3.0 (2.5–3.7)	2.7 (2.3–3.8)	0.07	2.38–4.98
D-dimer, ng/mL	0.38 (0.21–0.90)	0.38 (0.21–0.89)	0.65 (0.20–1.47)	0.05	0–0.50
Leucocyte count, × 10^9^/L	5.7 (4.7–6.9)	5.7 (4.7–6.9)	5.7 (4.9–7.6)	0.35	3.5–9.5
Neutrophil count, × 10^9^/L	3.3 (2.5–4.3)	3.3 (2.5–4.2)	3.4 (2.6–5.2)	0.24	1.8–6.3
Lymphocyte count, × 10^9^/L	1.6 (1.2–2.0)	1.6 (1.3–2.0)	1.6 (1.1–2.0)	0.51	1.1–3.2
Erythrocyte count, × 10^9^/L	4.1 (3.8–4.5)	4.1 (3.8–4.5)	4.2 (3.6–5.0)	0.20	4.3–5.8
Hemoglobin, g/L	126.0 (115.0–137.0)	126.0 (115.0–137.0)	128.0 (111.0–144.0)	0.59	130.0–175.0
Platelet count, × 10^9^/L	229.0 (187.0–277.2)	230.0 (189.0–278.0)	195.0 (148.5–265.5)	0.001	125.0–350.0
IgM of SARS-CoV-2	219 (35.7)	209 (35.6)	10 (37.0)	0.88	
IgG of SARS-CoV-2	531 (91.6)	508 (91.5)	23 (92.0)	1.00	

**Table 3:** Comorbidities in 1,788 patients with COVID-19.

**Comorbidity**	**Total (*n* = 1,788)**	**Non-elevated STB group (*n* = 1,719)**	**Elevated STB group (*n* = 69)**	***P*-value**	**Non-elevated CB group (*n* = 1,686)**	**Elevated CB group (*n* = 102)**	***P*-value**
Cardiovascular disease	356 (19.9)	340 (19.8)	16 (23.2)	0.87	326 (19.3)	30 (29.4)	0.01
Pulmonary disease	89 (5.0)	86 (5.0)	3 (4.3)	0.74	82 (4.9)	7 (6.9)	0.37
Nervous system disease	56 (3.1)	54 (3.1)	2 (2.9)	0.76	52 (3.1)	4 (3.9)	0.64
Endocrine disease	137 (7.7)	132 (7.7)	5 (7.2)	0.66	125 (7.4)	12 (11.8)	0.11
Malignancy	64 (3.6)	61 (3.5)	3 (4.3)	0.89	59 (3.5)	5 (4.9)	0.46
Digestive system disease	45 (2.5)	40 (2.3)	5 (7.2)	0.37	39 (2.3)	6 (5.9)	0.03

### Bilirubin Levels and Mortality

The association between bilirubin level and mortality is shown in Table 4. The elevated STB group had higher CB and UCB levels than the non-elevated STB group (both *P* < 0.001). Mortality was considerably higher in the elevated STB group (5.8%) than in the non-elevated STB group (0.6%, *P* < 0.001). Patients with elevated CB levels exhibited a similar result, while no deaths occurred in the elevated UCB group. The dynamic changes after the onset of increased bilirubin levels have been depicted using curve fitting analysis in Supplementary Figure 1.

**Table 4:** Bilirubin levels and mortality in 1,788 patients with COVID-19.

**Covariate**	**Total (*n* = 1,788)**	**Non-elevated STB group (*n* = 1,719)**	**Elevated STB group (*n* = 69)**	***P*-value**	**Mortality**	**Reference range**
DB/IB	0.53 (0.44–0.71)	0.53 (0.44–0.70)	0.55 (0.43–0.96)	0.29		
Serum total bilirubin, μmol/L	9.1 (7.0–12.0)	9.0 (6.9–11.6)	26.0 (22.8–31.1)	<0.001		5.0–21.0
5.0–21.0	1,595 (89.2)	1,595 (92.8)	0	<0.001	10/1,585	
<5.0	124 (6.9)	124 (7.2)	0		1/124	
>21.0	69 (3.9)	0	69 (100.0)		4/69	
Conjugated bilirubin, μmol/L	3.1 (2.4–4.3)	3.1 (2.4–4.1)	9.2 (7.5–14.0)	<0.001		0–7.0
0–7.0	1,686 (94.3)	1,675 (97.4)	11 (15.9)	<0.001	10/1,686	
>7.0	102 (5.7)	44 (2.6)	58 (84.1)		5/102	
Unconjugated bilirubin, μmol/L	5.7 (4.3–7.8)	5.6 (4.2–7.5)	16.1 (13.6–20.3)	<0.001		1.5–18.0
1.5–18.0	1,590 (98.2)	1,547 (99.4)	43 (68.3)	<0.001	13/1,590	
<1.5	10 (0.6)	9 (0.6)	1 (1.6)		1/10	
>18.0	19 (1.2)	0	19 (30.2)		0	
Mortality	15 (0.8)	11 (0.6)	4 (5.8)	<0.001		

### Survival Analyses

**Figure 1:**
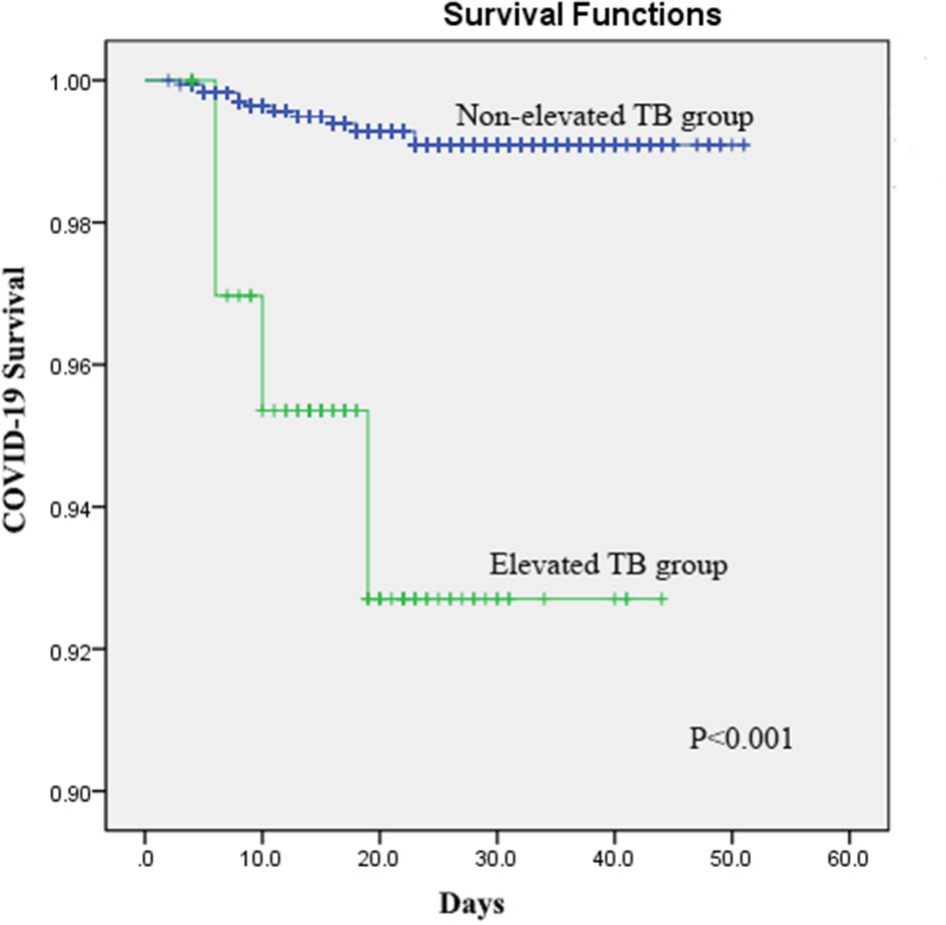
Kaplan–Meier curves for survival in patients with COVID-19 according to elevated serum total bilirubin (STB) levels.

The Kaplan–Meier analyses revealed that the elevated STB group had significantly higher mortality than the non-elevated STB group (*P* < 0.001, Figure 1). In the univariate Cox regression analyses, STB levels, CB levels, and CB/UCB were found to be related with COVID-19 survival. After adjusting for age, history of cardiovascular disease, leucocyte count, platelet count, lymphocyte count, and creatinine levels, multivariate Cox regression analysis revealed that patients with elevated STB levels had a significantly higher risk for COVID-19-related mortality [hazard ratio (HR): 9.45, 95% confidence interval (CI): 2.21–40.47, *P* = 0.002]. Elevated CB (HR: 4.38, 95% CI: 1.78–16.29, *P* = 0.03) and CB/UCB (HR: 2.49, 95% CI: 1.32–4.71, *P* = 0.01) were also regarded as potential factors influencing survival. Univariate and multivariate analyses showed that survival did not differ between patients with elevated and non-elevated UCB levels (both *P* > 0.05). The results are shown in Table 5.

**Table 5:** Univariate and multivariate Cox regression analyses for mortality in patients with COVID-19.

	**Group**	**HR**	**95% CI**		***P*-value**
**Cox regression analysis**					
Univariate analysis	Non-elevated STB	ref			
	Elevated STB	9.02	2.87	28.32	<0.001^∧^
Multivariate analysis^*^	Non-elevated STB	ref			
	Elevated STB	9.45	2.21	40.47	0.002^∧^
Univariate analysis	Normal CB	ref			
	Elevated CB	8.37	2.86	24.5	<0.001^∧^
Multivariate analysis^*^	Normal CB	ref			
	Elevated CB	4.38	1.78	16.29	0.03^∧^
Univariate analysis	Non-elevated UCB	ref			
	Elevated UCB	0.05	<0.001	1.80E+08	0.79
Multivariate analysis^*^	Non-Elevated UCB	ref			
	Elevated UCB	<0.001	<0.001	/	0.99
Univariate analysis	CB/UCB	4.42	2.64	7.42	<0.001^∧^
Multivariate analysis^*^	CB/UCB	2.49	1.32	4.71	0.01^∧^

* *Adjusted for age, history of cardiovascular disease, leucocyte count, platelet count, lymphocyte count, and creatinine level*.

*/Limit value*.

∧ *P < 0.05*.

### Association Between CB/UCB and Disease Severity

**Figure 2:**
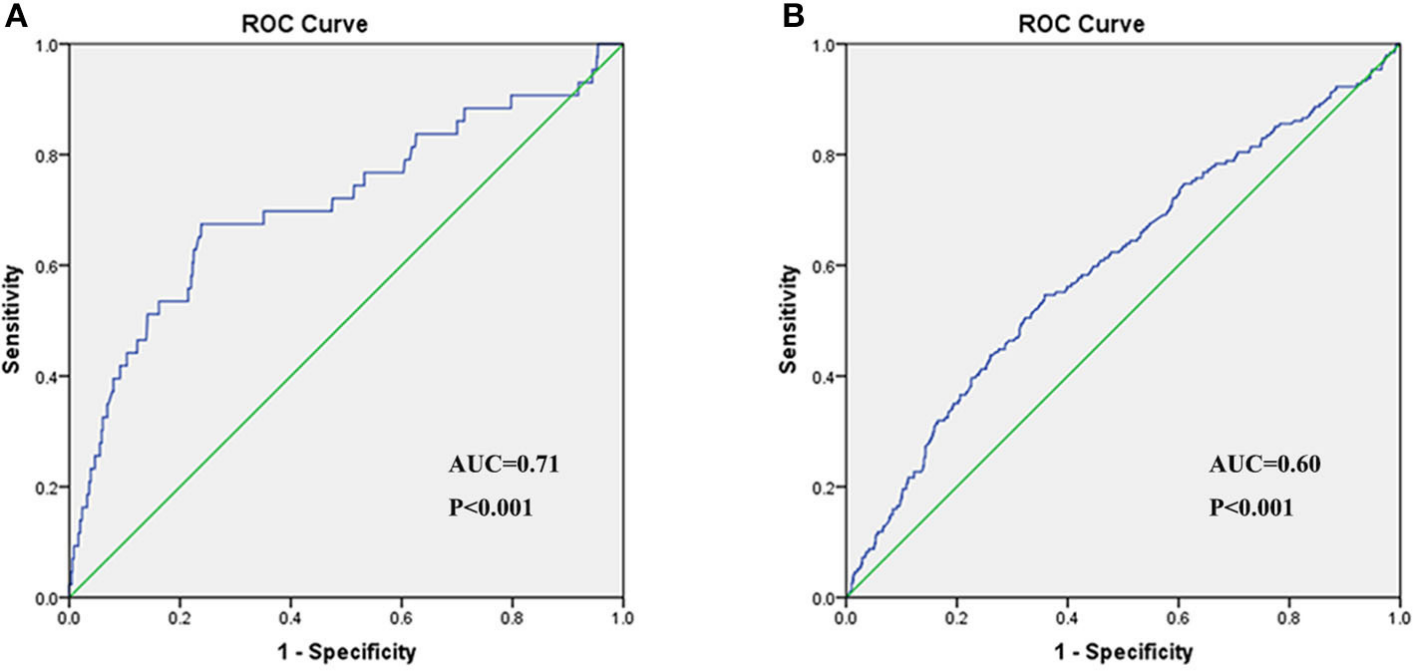
Receiver operating characteristic (ROC) curve of CB/UCB for disease severity **(A)** and hospitalization length **(B)** in patients with COVID-19.

In the univariate logistic regression analysis, the risk for critical disease increased with CB/UCB (Table 6). We found a similar trend in multivariate analysis [odds ratio (OR): 2.21, 95% CI: 1.20–4.07, *P* = 0.01, Table 6). The area under the ROC curve (AUC) of CB/UCB for critical disease was 0.71 (*P* < 0.001, Figure 2A). Therefore, there was a relation between CB/UCB and critical disease. According to ROC curve analysis, CB/UCB showed a correlation with hospitalization length >30 days (AUC: 0.60, *P* < 0.001, Figure 2B).

**Table 6:** Univariate and multivariate logistic regression analysis for critical disease in patients with COVID-19.

	**Group**	**OR**	**95% CI**		**P value**
**Logistic regression analysis**					
Univariate analysis	CB/UCB	4.42	2.63	7.42	< 0.001^∧^
Multivariate analysis^*^	CB/UCB	2.21	1.2	4.07	0.01^∧^

* *Adjusted for age, history of cardiovascular disease, leucocyte count, platelet count, lymphocyte count, and creatinine levels*.

∧ *P < 0.05*.

### Evaluation of CT Images

**Figure 3:**
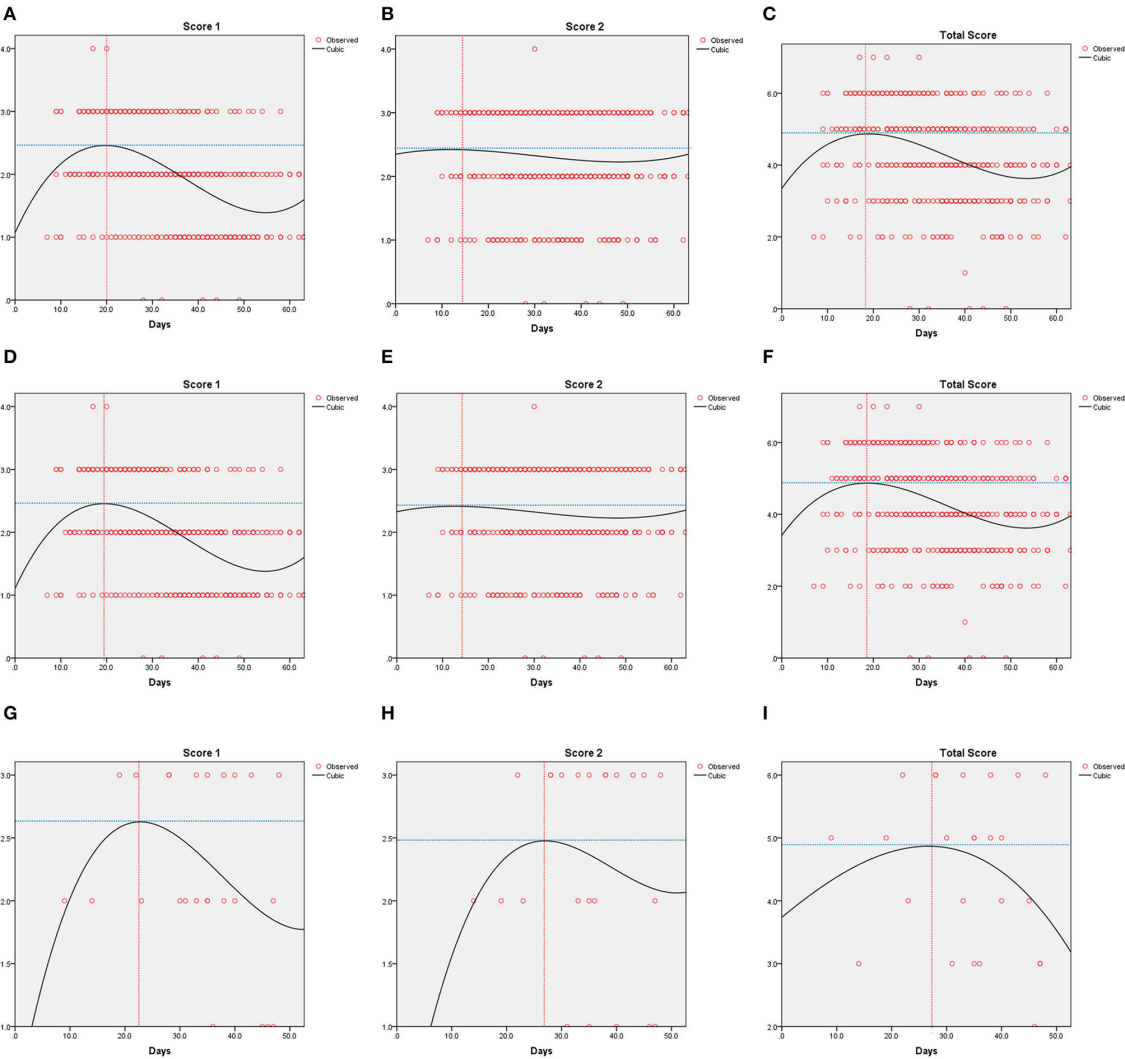
Curve fitting analysis for all patients **(A–C)**, the normal or decreased serum total bilirubin (STB) group **(D–F)** and the elevated STB group **(G–I)** in patients with COVID-19.

The results of curve fitting analyses are given in Figure 3. The peak of Score 1 was 2.5 on Day 20 for all patients, 2.5 on Day 19 for patients with non-elevated STB levels, and 2.6 on Day 23 for patients with elevated STB levels. Score 2 of all patients reached the peak on Day 14 (Score = 2.4), and the peak for patients with non-elevated STB levels was 2.4 on Day 14. However, Score 2 in patients with elevated STB levels showed a significant delay with regard to the inflection point (2.5 on Day 27). For all patients (4.9) and patients with non-elevated STB levels (4.9), the total score reached the peak on Day 18. Patients with elevated STB levels reached a delayed inflection point (4.9) on Day 27.

## Discussion

Based on our clinical experience during the outbreak of COVID-19, a small group of patients experienced increased bilirubin levels, regardless of the presence of pre-existing liver diseases. Patients with elevated bilirubin tended to have worse prognoses and more severe disease. Therefore, we conducted this study to determine whether bilirubin levels in COVID-19 patients were related to disease progression and prognosis. We found that 5.8% of patients in the elevated STB group died, compared to 0.6% of patients in the normal STB group. In the Cox regression analyses, those with elevated STB levels had a higher risk of mortality (unadjusted and adjusted). Results of the analyses of CB and CB/UCB showed a similar trend. However, UCB levels were not significantly associated with the survival of COVID-19 patients in either the univariate or multivariate Cox regression analyses. Univariate and multivariate logistic regression analyses demonstrated that the risk of critical infection increased with the elevation of CB/UCB. We used ROC curves to confirm the existence of a relationship between CB/UCB and disease severity. The ROC curves also showed a relation between CB/UCB and hospitalization length. Curve fitting analyses revealed that lung involvement progressed initially in all patients and then improved. However, patients with elevated STB levels showed a delay in reaching the turnaround point.

Angiotensin converting enzyme 2 (ACE2) receptor has been discovered to be a necessary entry receptor of SARS-CoV-2 in previous studies, which presents a wide distribution (16, 17). COVID-19 may affect many organs including the liver. SARS-CoV-2, the virus that causes COVID-19, is reported to share 82 and 50% of its genome with SARS-CoV and Middle East respiratory syndrome (MERS)-CoV, respectively. SARS-CoV and MERS-CoV both cause severe respiratory symptoms (18–20). Coincidentally, liver impairment has been reported in up to 60% of patients with SARS and also in patients with MERS (21, 22). Free bilirubin is derived from catabolism of heme, predominantly hemoglobin heme (23). Once produced, free bilirubin quickly binds to albumin, and this complex of free bilirubin bound to albumin in blood is called UCB. Therefore, free bilirubin only represents the fraction of bilirubin that remains unbound to any solubilizing compound in the vascular bed. Then, UCB enters the liver along with the blood stream and is rapidly absorbed by hepatocytes after isolated from albumin. After that, UCB turns into CB in hepatocytes through a sequence of biochemical reactions and CB is excreted into the bile ducts. So, an elevated CB level can be an important manifestation of liver injury.

Gong et al. demonstrated that patients with severe COVID-19 tended to have higher bilirubin levels. In another study, Wang et al. found a significantly higher level of bilirubin in intensive care unit (ICU) patients with COVID-19 than in non-ICU patients. Their results were consistent with our findings. An elevated bilirubin level is regarded as a vital marker of altered liver function, indicating liver damage. Chai et al. (24) demonstrated that the expression of ACE2 receptors in bile duct epithelial cells was relatively high and even equivalent to that in alveolar type II cells. However, hepatocytes may express ACE2 receptors at only one-twentieth the concentration found in bile duct epithelial cells. These findings suggest that epithelial cell damage in the bile duct may represent another mechanism of liver tissue injury, besides the direct infection of hepatocytes by SARS-CoV-2.

Due to the metabolic pathway of bilirubin, elevated UCB is common in hemolytic disease while elevated CB is usually related to damage to hepatocytes. Elevation of CB/UCB may indicate acute hepatitis. Thus, it is understandable that elevated STB levels, CB levels, and CB/UCB are associated with disease progression and prognosis. However, an increased UCB concentration is not only a marker of hemolysis but may also indicate a disorder of the bilirubin metabolism within the liver tissue. This parameter is probably associated with disease progression, but we did not obtain statistically significant results for the association between UCB concentration and COVID-19 survival. Further studies may be needed in this regard.

The study has several limitations. First, although 1,788 patients were enrolled in the study, there were only 69 patients in the elevated STB group. Second, some medical data which available in some patients and missing in other patients may exist some contrary effect on our primary outcomes. Third, owing to the retrospective nature of the study, we could not avoid sample heterogeneity and some parameters associated with disease prognosis were not recorded in the medical record system. Fourth, absence of data on cholestatic enzymes and data on pre-hospitalization bilirubin was a great pity. Fifth, only 4 patients died in the elevated STB group, thus the number is low and further larger epidemiological studies are required. Finally, Gilbert syndrome, known as a common genetic unconjugated bilirubinemia, may confound the possible association of bilirubin and severity of COVID-19 disease. Therefore, more attention should be focused on this issue in the future, and multicenter studies with a bigger sample is needed. Moreover, continuously monitoring to the changes of bilirubin levels may be of great value.

In conclusion, elevated STB levels, CB levels, and CB/UCB were associated with a higher risk of COVID-19 mortality. Moreover, CB/UCB was associated with disease severity and length of hospitalization; thus, it may be useful as a prognostic indicator. Patients with elevated STB levels tended to have more severe pneumonia and took longer to recover than patients with normal STB; thus, it is necessary to pay special attention to COVID-19 patients with elevated bilirubin levels in clinical management.

## Data Availability Statement

The raw data supporting the conclusions of this article will be made available by the authors, without undue reservation.

## Author Contributions

ZLiu, JL and LG contributed conception and design of the study. WL performed the statistical analysis and wrote the first draft of the manuscript. WZ, RG, ZLi and XW contributed to data acquisition. GZ, DC, SW, QL and DH contributed to manuscript revision. All authors contributed to data interpretation and approved the final version.

## Conflict of Interest

The authors declare that the research was conducted in the absence of any commercial or financial relationships that could be construed as a potential conflict of interest.

## Supplementary Material

Supplementary Figure 1 Curve fitting analysis for the dynamic changes of bilirubin in patients with COVID-19.

## Abbreviations

COVID-19: The coronavirus disease
ROC: Receiver operating characteristic
STB: Serum total bilirubin
HR: Hazard ratio
CB: Conjugated bilirubin
UCB: Unconjugated bilirubin
CB/UCB: Conjugated bilirubin to unconjugated bilirubin
SARS: Severe acute respiratory syndrome
SARS–CoV−2: Severe acute respiratory syndrome coronavirus 2
CT: Computed tomography
GGO: Ground–glass opacities
IQR: Interquartile range
ECMO: Extracorporeal membrane oxygenation
INR: International normalized ratio
CI: Confidence interval
OR: Odds ratio
ACE2: Angiotensin converting enzyme 2
MERS: Middle East respiratory syndrome
ICU: Intensive care unit.
