# Supplementary figures and images for: Bilirubin Levels as Potential Indicators of Disease Severity in Coronavirus Disease Patients: A Retrospective Cohort Study
Source: Front Med (Lausanne). 2020 Nov 9;7:598870. doi: 10.3389/fmed.2020.598870 (PMC7680876; doi:10.3389/fmed.2020.598870)

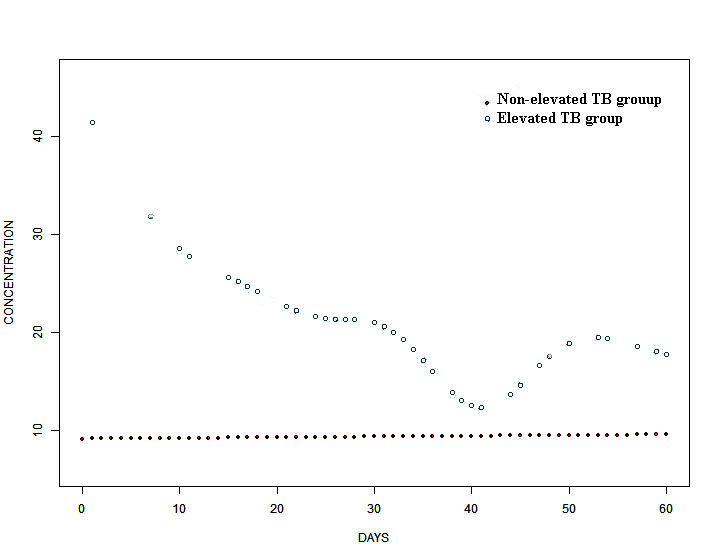

Supplement: Supplementary Figure 1 — Curve fitting analysis for the dynamic changes of bilirubin in patients with COVID-19. [file Image_1.JPEG]
